# Evaluation of the Early Intervention Physiotherapist Framework for Injured Workers in Victoria, Australia: Data Analysis Follow-Up

**DOI:** 10.3390/healthcare11152205

**Published:** 2023-08-04

**Authors:** Hadi Akbarzadeh Khorshidi, Uwe Aickelin, Andrea de Silva

**Affiliations:** 1 Melbourne School of Population and Global Health, The University of Melbourne, Melbourne 3052, Australia; 2 School of Computing and Information Systems, The University of Melbourne, Melbourne 3052, Australia; uwe.aickelin@unimelb.edu.au; 3 School of Public Health and Preventive Medicine, Monash University, Melbourne 3800, Australia; andrea.desilva@monash.edu

**Keywords:** early intervention, return to work, physiotherapists, workers’ compensation, evaluation

## Abstract

Purpose: This study evaluates the performance of the Early Intervention Physiotherapist Framework (EIPF) for injured workers. This study provides a proper follow-up period (3 years) to examine the impacts of the EIPF program on injury outcomes such as return to work (RTW) and time to RTW. This study also identifies the factors influencing the outcomes. Methods: The study was conducted on data collected from compensation claims of people who were injured at work in Victoria, Australia. Injured workers who commenced their compensation claims after the first of January 2010 and had their initial physiotherapy consultation after the first of August 2014 are included. To conduct the comparison, we divided the injured workers into two groups: physiotherapy services provided by EIPF-trained physiotherapists (EP) and regular physiotherapists (RP) over the three-year intervention period. We used three different statistical analysis methods to evaluate the performance of the EIPF program. We used descriptive statistics to compare two groups based on physiotherapy services and injury outcomes. We also completed survival analysis using Kaplan–Meier curves in terms of time to RTW. We developed univariate and multivariate regression models to investigate whether the difference in outcomes was achieved after adjusting for significantly associated variables. Results: The results showed that physiotherapists in the EP group, on average, dealt with more claims (over twice as many) than those in the RP group. Time to RTW for the injured workers treated by the EP group was significantly lower than for those who were treated by the RP group, indicated by descriptive, survival, and regression analyses. Earlier intervention by physiotherapists led to earlier RTW. Conclusion: This evaluation showed that the EIPF program achieved successful injury outcomes three years after implementation. Motivating physiotherapists to intervene earlier in the recovery process of injured workers through initial consultation helps to improve injury outcomes.

## 1. Introduction

Injuries or illnesses occurring at work have a substantial impact on individuals and society [1]. Although most injured workers can successfully recover and achieve a return to work (RTW) [2], RTW may take a longer time for many injured workers. RTW is a complicated process and can be impacted by different factors, including physical, psychological, social, and policy-related ones. There are several stages for an injured worker to get ready for RTW [3]. As the time that an injured worker is away from work gets longer, the likelihood of not returning to work increases [4]. Therefore, speedy and sustained RTW is the main goal of compensable injury systems, including those in Victoria, Australia, such as WorkSafe Victoria (WSV) and Transport Accident Commission (TAC). To reach this goal, early identification of undesired outcomes [5], such as delayed RTW, and early intervention [6] to prevent the undesired outcomes would be beneficial. 

Physiotherapists and their early intervention play an important role in occupational rehabilitation [7] and, consequently, in reducing the post-injury cost [8] and improving RTW [9]. Also, the prominence of physiotherapy in treating musculoskeletal injuries is well recognized. However, the timing of the commencement of physiotherapy treatment, in other words, the time of physiotherapists’ intervention, is less clear [10]. Therefore, WSV, in collaboration with TAC, implemented the Early Intervention Physiotherapist Framework (EIPF) in 2014. The EIPF aimed to encourage physiotherapists who were working with compensable clients to work with clients early in the treatment program in relation to their RTW. The EIPF was implemented through an online training program for physiotherapists. This Framework was designed to encourage physiotherapists to engage clients in physical therapies early in the Occupational Rehabilitation (OR) process, with the aim of decreasing time to RTW and improving RTW sustainability. Once the program was successfully completed and a physiotherapist was accredited, higher fees were paid for services provided by these EIPF physiotherapists to encourage participation. WSV also provided incentives for physiotherapists who had their initial consultation for injured workers within 7 months post-injury [11].

An initial evaluation was undertaken immediately post-EIPF program delivery [12]. However, the duration of this evaluation was too short (3 months) to identify data trends and determine the impact of the program on RTW outcomes. In [12], the authors suggested that another evaluation should be undertaken with a sufficient follow-up period. In the current study, we initiated the second evaluation to examine the performance of the EIPF program three years after implementation in 2017. We also studied the differences in physiotherapy services and RTW outcomes for injured workers to examine the impact of the EIPF program on WSV clients.

### 1.1. Aims 

This study aims to determine if the implementation of the EIPF program is associated with differences in the physiotherapy services provided or RTW outcomes for injured workers. 

The study examines the following research questions: 

RQ1. Are there differences in physiotherapy services provided to WSV clients between EIPF-trained physiotherapists (EP) and regular physiotherapists (RP) over the three-year intervention period (2014–2017)?

- Specifically, the type and number of services as well as the frequency of claims.

RQ2. Are there differences in the RTW outcomes of WSV clients treated by either EP or RP over the three-year intervention period (2014–2017)?

- Specifically, any difference between the time taken from the injury date to the RTW date, adjusting for explanatory variables (age, gender, type of injury, and occupation) as required.

The time to RTW is a common measure to evaluate the performance and success of injury intervention and healthcare improvement programs [13,14]. 

### 1.2. Definitions

The following definitions are used in the data analysis:

- EIPF—Early Intervention Physiotherapy Framework program (the intervention)
- EP—Physiotherapists who completed the EIPF program
- RP—Regular physiotherapists who did not complete the EIPF program
- WSV—WorkSafe Victoria.

## 2. Materials and Methods

To evaluate differences between types of physiotherapists and the effectiveness of the EIPF on client outcomes (addressing the research questions), a comparison of EP and RP was made. To assess the impact of the EIPF program, only claims which were served exclusively by physiotherapists of either the EP or RP group were analyzed. The manuscript complies with Transparent Reporting of a multivariable prediction model for Individual Prognosis Or Diagnosis (TRIPOD) statement [15] using the TRIPOD checklist that is presented in Table 1.

### 2.1. Data

The source of data is the Compensation Research Database (CRD) which was held by the Institute for Safety, Compensation and Recovery Research (ISCRR). The CRD includes the details of all claims, payments, services, hospital admissions, and medical certificates for WSV since 1985. It is an administrative database that is fully de-identified, and consent to use data for research purposes is obtained from clients [16,17]. The selection criteria for claims included in this study are as follows:

- Claims that had at least one day of wage compensation payment (standard time loss claims) with the injury date on or after the first of January 2010,
- Claims that had physiotherapy services provided either by EP or RP,
- Claims that had their initial physiotherapy consultation on or after the first of August 2014,
- If claims resulted in RTW, only claims with RTW on or after the date of initial consultation were included.

According to the selection criteria, 17,991 claims were identified, from which 7363 claims were served only by the EP group, 3998 claims were served only by the RP group, and 6630 claims were served by a mix of EP and RP groups. To provide a better investigation of the performance of the EIPF program, the claims in the mixed group were excluded from further analysis.

### 2.2. Outcomes

Time to RTW is calculated as the number of days between the injury date and the resumed work date. Comparing this outcome between two groups allows for an analysis of the effectiveness of the EIPF on client RTW outcomes.

### 2.3. Analysis

Different data analysis approaches have been used to answer the research questions. We used descriptive statistics to comprehensively compare the differences between the EP and RP groups in terms of physiotherapy services provided to clients and time to RTW. 

We performed survival analysis using Kaplan–Meier curves to estimate the probability of RTW in each time interval. Kaplan–Meier curves are commonly used tools to analyze ‘time-to-event’ data [18]. These have been used widely in healthcare areas such as job survival of impaired employees [19] and vision loss after Diabetic Vitrectomy surgery [20]. In this study, we used the time to RTW in survival analysis. 

We also used the log-rank test, which is a non-parametric test, to compare survival curves between two groups. The log-rank test, like the Kaplan–Meier curves, is used to compare two groups, e.g., treated versus the control group in a randomized trial. Also, the follow-ups can be divided into smaller time periods, and the number of occurrences within all time periods is compared. Similar to the Kaplan–Meier curves, the log-rank test should be used only when follow-ups are reasonably current. The log-rank test is limited to assessing the effect of just one variable at a time. A more complex method, such as the Cox model, should be considered for assessing multiple variables [21].

We assessed the association between time to RTW and claimants’ characteristics as predictors using univariate regression analysis. These predictors are gender, age, type of injury, and occupation. We also used multivariate regression to determine whether there is a difference in RTW outcomes achieved after adjusting for variables that are significantly associated with the RTW outcome.

## 3. Results

This section presents the results of data analysis on the evaluation of the EIPF program across EP and RP groups.

### 3.1. Descriptive Statistics

#### 3.1.1. Description of Dataset

Table 2 summarizes the claimants’ characteristics for injured workers in the EP and RP groups. All percentages are out of the total population of each group. For age groups, our groups are aligned with the relevant studies derived from CRD [22]. For injury types and occupation groups, we kept the WSV-defined categories. The results show that the groups were not statistically different in terms of client age, gender, injury type, or occupation. This provides a fair baseline from which to compare the two groups based on physiotherapy services.

#### 3.1.2. Services and Claims

In this section, we compare two groups in terms of physiotherapy services provided to clients (addressing RQ1). Physiotherapists use a variety of techniques (or services) to treat their clients and support them with RTW after injury. The average services per claim and average number of claims for each physiotherapist type (even if there were multiple physiotherapists servicing the claim) are shown in Table 3.

The average number of claims each physiotherapist treated was significantly higher in the EP group than the RP one. In the EP group, each physiotherapist treated about 10 claims, whereas, in the RP group, each physiotherapist only treated 4–5 claims over the three-year period. The average number of services provided per claim was not different between the two groups.

#### 3.1.3. Time to Achieving RTW

In this section, we evaluate differences between EP and RP groups based on time to RTW (addressing RQ2). Table 4 shows the results for the days taken from the injury date to the RTW date.

The results show that the time from the date of injury to the RTW date was significantly shorter (on average, about 3 weeks shorter) for clients treated by the EP group compared to the RP group.

#### 3.1.4. Proportion Achieving RTW

In this section, we evaluate the difference between EP and RP groups based on the proportion of claims that had RTW for the periods of the whole three years and six months after injury. Table 5 below shows the RTW status of injured workers treated by the EP and RP groups over the full three-year evaluation period and at six months post-injury.

The proportion of claims that had RTW was not different between the EP and RP groups overall; however, at 6 months post-injury, significantly more clients in the EP group had returned to work than the RP group. We will investigate this in more detail in the Survival Analysis.

### 3.2. Survival Analysis

**Figure 1:**
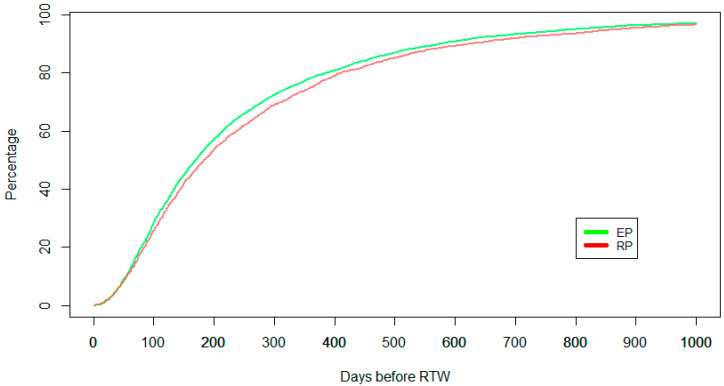
Survival analysis for time to RTW from injury date over three years.

In this section, we perform survival analysis based on time to RTW (addressing RQ2). Figure 1 shows the Kaplan–Meier curves for EP and RP groups up to 1000 days post-injury (about three years). The Kaplan–Meier curves show that patients who have been served by physiotherapists who completed the EIPF program (EP) returned to work earlier than patients who visited regular physiotherapists (RP). We examine the significance of the difference between curves statistically using a log-rank test. The log-rank test shows that the difference in the time to RTW was statistically significant by physiotherapist type (EP vs. RP; χ2 statistic 10.58, *p*-value < 0.001) for all claims.

Table 6 presents the percentages of RTW and outputs of a log-rank test to examine the difference of Kaplan–Meier curves in the periods of 3, 6, 12, 24, and 36 months. It shows that the difference in the time to RTW becomes significant for clients who achieved their RTW in 2 years (χ2 statistic 6.94, *p*-value < 0.001) and in 3 years (χ2 statistic 12.1, *p*-value < 0.001) post-injury. Considering the results, the effectiveness of the EIPF program has been shown after 2 years (within 24 to 36 months). It supports the follow-up period of three years that has been taken for this study and suggested by [12].

### 3.3. Regression Analysis

Regression analysis examines the association between time to RTW and the claimant’s characteristics. Only clients that were treated by either EP or RP groups were included, and both univariate and multivariate regression were used to examine the relationship between predictors and RTW outcomes. We use linear regression analysis. In the univariate regression model, each variable is the only predictor to predict the number of days to achieve RTW, and the *p*-value is calculated based on the *t*-test. In the multivariate regression model, all variables are included as predictors, and the *p*-value is calculated based on an F-test.

#### 3.3.1. Univariate Analysis

Table 7 shows that age and injury type were significant predictors in determining RTW outcome. However, gender and occupation were not. There was also a significant difference in RTW outcomes by the physiotherapy group, with those in the EP group returning to work 21 days faster than the RP group.

#### 3.3.2. Multivariate Analysis

We created a multivariate model to perform the regression analysis. The multivariate model adjusted the time to RTW for age and injury type, given the observed statistical association, and examined differences between the outcome between EP and RP groups. A significant difference remained, with injured workers in the EP group still RTW 21 days faster than those in the RP group (Table 7).

## 4. Discussion

The objective of this study was to describe the outcomes of an evaluation of the effects of the Early Intervention Physiotherapist Framework (EIPF) program on the return to work (RTW) outcomes for injured Victorian workers. The RTW outcomes were assessed three years after the initial implementation of the EIPF, and the effects on clients were examined in comparison with clients treated by physiotherapists not trained in the EIPF (RP group).

Within the examination period, comparing physiotherapists with EIPF training (EP group) to the RP group revealed the following results:

- Physiotherapists in the EP group visited more WSV clients per physiotherapist (average of 10.2 claims) than those in the RP group (average of 4.6 claims) over the three-year period.
- Injured workers returned to work on average 25 days sooner when treated by EP compared with RP. This could be a direct result of EIPF by motivating physiotherapists (EP group) for earlier post-injury intervention.
- Survival analysis showed that the clients of the EP group returned to work significantly faster than the RP group. After two years, this difference became statistically significant, as shown by the log-rank test, confirming the necessity of a three-year follow-up analysis such as that performed in this study.
- The time to return to work was significantly associated with age and injury type in both physiotherapy groups. Younger injured workers (between 15 and 24 years old) with fractures had returned to work faster, and the injured workers between 45 and 54 years old with musculoskeletal injuries had taken more time to get back to work.
- After adjusting age and injury type variables, injured workers in the EP group still returned to work significantly faster than those treated in the RP group (by 21 days). These results show that the difference between these two groups was substantially associated with the early intervention by EP physiotherapists post-injury.

The results support the positive findings of the initial study [12] on the EIPF program (3 months after initiating the program) in terms of adhering to EIPF goals and RTW outcomes.

### Strengths and Limitations

The main strength of this study is the use of a well-structured and population-based dataset to evaluate the performance of the early intervention program. The findings result in an insightful conclusion on the important factors that affect RTW outcomes.

The main limitation of this study is that the data are for injured workers in Victoria, Australia. Therefore, the results cannot be generalized to every group of patients. The data we used in this study are administrative and payment data, which meant we relied on wage compensation to define RTW. Also, because the payment date for the physiotherapy services could be different from the actual date of receiving the service, the time to the first consultation session cannot be reliably calculated. Hence, we did not include this indicator in the analyses.

## 5. Conclusions

This evaluation has shown that three years after implementation, the EIPF resulted in positive outcomes for injured workers. More claims were managed by physiotherapists trained in EIPF than non-EIPF physiotherapists. The earlier intervention by physiotherapists (possibly within seven months after the injury) due to the incentives and the increased reimbursement provided by WSV for services led to a faster return to work and better outcomes for the injured worker.

The EIPF is achieving its aim of focusing on early intervention and sustainable return to work. Further monitoring of outcomes and performance will be important to ensure gains continue to be made on the time taken to the initial consultation post-injury, as it seems that any small improvement in this aspect can have a significant impact on the RTW outcomes of injured workers.

Further monitoring of outcomes and performance will be important to ensure gains continue to be made on the time taken to the initial consultation post-injury, as it seems that any small improvement in this aspect can have a significant impact on the RTW outcomes of injured workers. Early intervention programs and follow-up studies can be used in other allied health professions like chiropractic and osteopathy that play similar roles to physiotherapists in treating injured workers. In addition, the quality of the prediction by the conducted regression analysis can be investigated for future cases.

## Figures and Tables

**Table 1:** TRIPOD checklist.

**Title and Abstract**		
1	Title	Our paper includes a title which reflects the aim of the study
2	Abstract	Our paper includes an abstract which provides a summary of background, objectives, study design, participants, predictors, outcomes, statistical analysis, results and conclusions.
**Introduction**		
3	Background and objectives	Our paper specifies the objectives, explains the background of the study and refers to existing studies.
**Methods**		
4	Source of data	Our paper describes the source of data and specifies the selection criteria.
5	Participants	Our paper specifies who participated in the study.
6	Outcome	Our paper defines the outcomes clearly.
7	Predictors	Our paper clearly explains all predictors used in analyses.
8	Sample size	Our paper mentions the sample size for both intervention and control groups.
9	Missing data	There were no missing data.
10	Statistical analysis methods	Our paper describes all the statistical analysis methods used in the study.
11	Risk groups	We did not have any risk group in our study.
12	Development vs validation	Our paper compares the distribution of categories in intervention and control groups.
**Results**		
13	Participants	Our paper includes descriptive statistics about participants.
14	Model development	Our paper compares two groups using descriptive statistics, survival analysis and regression models.
15	Model specification	Our paper reports all parameters associated with the statistical analysis methods.
16	Model performance	Our paper presents the results of statistical tests for comparison.
17	Model updating	Our paper explains how we adjusted for the impact of other predictors on the comparison.
**Discussion**		
18	Limitations	Our paper lists limitations and refers to potential future work to address these.
19	Interpretation	Our paper discusses the results and interprets the outcomes considering the research questions and goals.
20	Implications	Our paper discusses how the results provide insights on the performance of an early intervention program.
**Other information**		
21	Supplementary Information	Our paper provides information about ethics.
22	Funding	Our paper acknowledges the source of funding for the study.

**Table 2:** Distribution of injured workers based on characteristics in the EP and RP groups.

Claimant Characteristics	EP		RP	
	Number	Percentage	Number	Percentage
**Gender**				
Male	4909	66.67%	2563	64.11%
Female	2454	33.33%	1435	35.89%
**Age groups**				
15–24	552	7.50%	298	7.45%
25–34	1269	17.23%	698	17.46%
35–44	1610	21.87%	803	20.09%
45–54	2120	28.79%	1104	27.61%
55–64	1622	22.03%	952	23.81%
Others	190	2.58%	143	3.58%
**Injury type**				
Fractures	1090	14.80%	594	14.86%
Joints	1887	25.63%	1062	26.56%
Mental	35	0.48%	16	0.40%
Musculoskeletal	3442	46.75%	1902	47.57%
Wounds	606	8.23%	241	6.03%
Other injuries	142	1.93%	98	2.45%
Other diseases	161	2.19%	85	2.13%
**Occupation groups**				
Managers	224	3.04%	161	4.03%
Professionals	659	8.95%	432	10.81%
Associate professionals	604	8.20%	387	9.68%
Tradespersons	1403	19.05%	694	17.36%
Advanced clerical and Service workers	84	1.14%	68	1.70%
Intermediate clerical, sales, and Service workers	817	11.10%	479	11.98%
Intermediate production and transport workers	1677	22.78%	741	18.53%
Elementary clerical, sales, and Service workers	288	3.91%	179	4.48%
Laborers	1607	21.83%	857	21.44%

**Table 3:** Distribution of injured workers based on characteristics in EP and RP groups.

Measures	EP		RP	
	Mean	95% CI	Mean	95% CI
Number of claims treated per physiotherapist	10.2 *	[9.6, 10.8]	4.6	[4.3, 4.9]
Physiotherapy services per claim	29.6	[28.9, 30.4]	28.5	[24.4, 29.6]

95% CI: 95% confidence interval; * significantly higher than RP (*p* < 0.0001).

**Table 4:** Time to RTW in EP and RP groups.

Measures	EP		RP		Difference (EP-RP)	*p*-Value *
	Mean	95% CI	Mean	95% CI		
Days from date of Injury to RTW	267.7	[259.3, 276.2]	289.1	[277.0, 301.2]	−25.4	**<0.01**

* Based on *t*-test of difference between groups.

**Table 5:** Proportion of RTW achieved by injured workers in the EP and RP groups.

RTW Status/Proportion	EP	RP
Three years		
RTW	70.4%	68.8%
RTW—full-time	55.8%	56.0%
RTW—part-time	14.6%	12.7%
No RTW	29.6%	31.2%
Six months		
RTW	37.5%	34.2%
RTW—full-time	30.9%	29.2%
RTW—part-time	6.7%	5.0%
No RTW	62.5%	65.8%

**Table 6:** Comparison of RTW percentage between EP and RP over time.

Time Period (Months)	RTW Percentage		Log-Rank Test	
	EP	RP	Chi-Square Stat	*p*-Value
3	23.90%	22.23%	0.25	0.62
6	53.31%	49.72%	0.22	0.64
12	78.53%	75.30%	2.83	0.09
24	93.69%	92.54%	6.94	**<0.001**
36	96.93%	96.51%	12.11	**<0.001**

**Table 7:** Regression analysis with the outcome being the number of days to achieving RTW.

	Univariate		Multivariate	
	Coefficient	Variable *p*-Value	Coefficient	Model *p*-Value
**Physiotherapy group**				**<0.0001**
RP	Ref	**<0.01**	Ref	
EP	−21.38		−21.57	
**Gender**				
Male	Ref	n.s.	-	
Female	4.77		-	
**Age groups**				
15–24	Ref	**<0.0001**	Ref	
25–34	26.39		14.60	
35–44	87.84		68.55	
45–54	93.62		78.58	
55–64	59.63		49.33	
Others	9.33		0.77	
**Injury type**				
Fractures	Ref	**<0.0001**	Ref	
Joints	53.34		51.80	
Musculoskeletal	179.22		159.78	
Mental	97.92		94.63	
Other Diseases	−22.47		−16.13	
Other Injuries	61.83		62.05	
Wounds	144.06		130.42	
**Occupation**				
Managers	Ref	n.s.	-	
Professionals	303.68		-	
Associate professionals	−35.51		-	
Tradespersons	−31.52		-	
Advanced clerical and service workers	−29.55		-	
Intermediate clerical, sales, and service workers	1.37		-	
Intermediate production and transport workers	−43.97		-	
Elementary clerical, sales, and service workers	−36.85		-	
Laborers	−8.73		-	

n.s. stands for not significant, and bold number indicates significance.

## Data Availability

Data cannot be available publicly.
